# Digital Technology Applications in the Management of Adverse Drug Reactions: Bibliometric Analysis

**DOI:** 10.3390/ph17030395

**Published:** 2024-03-19

**Authors:** Olena Litvinova, Andy Wai Kan Yeung, Fabian Peter Hammerle, Michel-Edwar Mickael, Maima Matin, Maria Kletecka-Pulker, Atanas G. Atanasov, Harald Willschke

**Affiliations:** 1 Department of Management and Quality Assurance in Pharmacy, National University of Pharmacy of the Ministry of Health of Ukraine, 61002 Kharkiv, Ukraine; 2 Ludwig Boltzmann Institute Digital Health and Patient Safety, Medical University of Vienna, 1090 Vienna, Austria; ndyeung@hku.hk (A.W.K.Y.); fabian.hammerle@meduniwien.ac.at (F.P.H.); maria.kletecka-pulker@univie.ac.at (M.K.-P.); 3 Oral and Maxillofacial Radiology, Applied Oral Sciences and Community Dental Care, Faculty of Dentistry, University of Hong Kong, Hong Kong, China; 4 Department of Anaesthesia, Intensive Care Medicine and Pain Medicine, Medical University of Vienna, 1090 Vienna, Austria; 5 Institute of Genetics and Animal Biotechnology of the Polish Academy of Sciences, 05-552 Magdalenka, Poland; michel.mickael77@gmail.com (M.-E.M.); m.matin@igbzpan.pl (M.M.); 6 Institute for Ethics and Law in Medicine, University of Vienna, 1010 Vienna, Austria

**Keywords:** patient safety, adverse drug reactions, drug discovery, preclinical studies, clinical trials, digital health, pharmacovigilance, artificial intelligence, machine learning

## Abstract

Adverse drug reactions continue to be not only one of the most urgent problems in clinical medicine, but also a social problem. The aim of this study was a bibliometric analysis of the use of digital technologies to prevent adverse drug reactions and an overview of their main applications to improve the safety of pharmacotherapy. The search was conducted using the Web of Science database for the period 1991–2023. A positive trend in publications in the field of using digital technologies in the management of adverse drug reactions was revealed. A total of 72% of all relevant publications come from the following countries: the USA, China, England, India, and Germany. Among the organizations most active in the field of drug side effect management using digital technologies, American and Chinese universities dominate. Visualization of publication keywords using VOSviewer software 1.6.18 revealed four clusters: “preclinical studies”, “clinical trials”, “pharmacovigilance”, and “reduction of adverse drug reactions in order to improve the patient’s quality of life”. Molecular design technologies, virtual models for toxicity modeling, data integration, and drug repurposing are among the key digital tools used in the preclinical research phase. Integrating the application of machine learning algorithms for data analysis, monitoring of electronic databases of spontaneous messages, electronic medical records, scientific databases, social networks, and analysis of digital device data into clinical trials and pharmacovigilance systems, can significantly improve the efficiency and safety of drug development, implementation, and monitoring processes. The result of combining all these technologies is a huge synergistic provision of up-to-date and valuable information to healthcare professionals, patients, and health authorities.

## 1. Introduction

The principle “First, do no harm” is a fundamental rule of medical ethics. Ensuring patient safety is a top priority for the World Health Organization. According to the World Health Organization (WHO), patient safety is defined as preventing and reducing the risk, errors, and extent of damage caused to patients in the process of providing health care [1]. The International Pharmaceutical Federation (FIP), focusing on the terminology of the Patient Safety Network (PSNet), defines patient safety as the absence of accidental harm arising from the provision of medical care, including preventable harm [2].

The development of modern methods for managing patient safety is inextricably linked to drug safety. According to terminology developed by WHO, harmful, unintended reactions to medicines that occur at doses normally used for treatment are called adverse drug reactions (ADRs) [3].

Adverse drug reactions are one of the most urgent problems in clinical medicine. The harms associated with medication use affect one in thirty patients seeking medical attention [4]. There is no doubt that the healthcare use of medicinal products should, first of all, be accompanied by a therapeutic effect, but the negative consequence of this process is the possibility of adverse reactions. According to various estimates, hospitalized patients in 5–8% of cases have adverse reactions to drugs [5,6]. The underestimation and belated solution to the problem of adverse drug reactions can result in the development of serious consequences. Unfortunately, in some cases, adverse reactions are fatal [7]. Treating adverse drug reactions causes additional financial costs [8]. This reduces the quality of medical care provided, and makes this problem not only medical but also economic and social. It is worth noting that the pediatric population is most frequently affected by drug complications [9].

A steady increase in the number of side effects of pharmacotherapy contributes to an increase in the number of allergic reactions due to contamination of the biosphere, use of pesticides, nitrates, preservatives, antibiotics, hormonal agents, polypharmacy, self-medication, etc. Making progress in current clinical practice depends on successfully addressing the question of the balance between efficacy and safety of drug treatment.

Versatile implementations of digital technologies are set to transform healthcare [10,11,12,13]. With the aim of improving patient safety, WHO has included the development of digital technologies in its Global Action Plan 2021–2030 [14].

In the field of drug development and implementation, as well as pharmacovigilance, the use of artificial intelligence, machine learning, and big data analysis provide ample opportunities. Artificial intelligence in general can be used to automate the processes of finding new drugs, and predicting and evaluating their effectiveness and safety. Machine learning can be used to analyze clinical data to identify potential interactions and personalize therapies. Analysis of big data in pharmacovigilance helps to quickly identify safety issues. These technologies increase the efficiency of drug development processes and ensure the safety and personalization of treatment.

The aim of this study is a bibliometric analysis of the use of digital technologies to prevent adverse drug reactions and an overview of their main directions to improve the safety of pharmacotherapy.

## 2. Results

**Figure 1:**
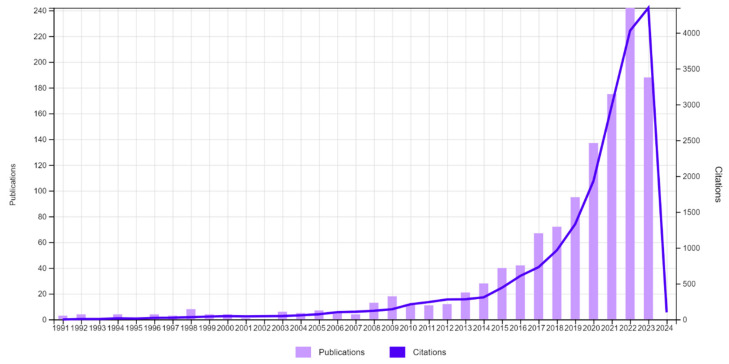
Dynamics of publications containing research keywords in the field of using digital technologies in the management of adverse drug reactions over the period 1991–2023.

An array containing 1241 documents was received as a result of the literature query conducted (as detailed in Section 4). An analysis of the dynamics of publications in the field of using digital technologies in the management of adverse drug reactions for the period from 1991 to 2023 indicates its positive, exponential nature (Figure 1).

The array of documents under consideration is presented in Table 1. The high share of articles is noteworthy, which testifies to the rapid scientific development of the field.

**Figure 2:**
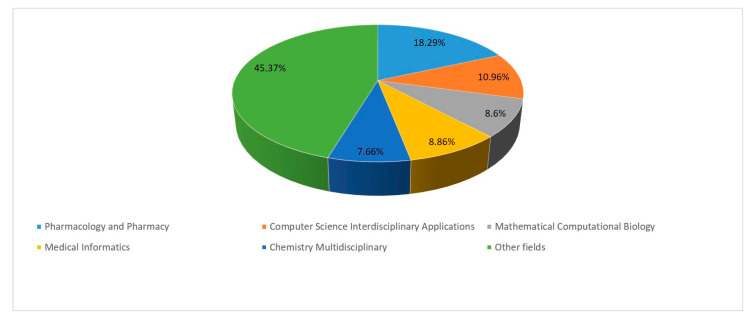
Distribution of publications containing research keywords by areas of knowledge for the period 1991–2023.

A total of 54.6% of scientific publications on the use of digital technologies in the management of adverse drug reactions cover the following areas of knowledge: Pharmacology and Pharmacy (18.29%), Computer Science Interdisciplinary Applications (10.96%), Mathematical Computational Biology (8.86%), Medical Informatics (8.86%), and Chemistry Multidisciplinary (7.66%) (Figure 2). The findings suggest that many scientific disciplines are widely used in research on the application of digital technologies in the management of adverse drug reactions, and an interdisciplinary approach is a key aspect in this area.

A wide geographic distribution of institutions conducting research on the topic under study was revealed. It was found that 72% of all publications come from the following countries: the USA, China, England, India, and Germany. Most of the reports (32%) are from the USA.

Table 2 shows the most productive organizations, journals, and publishers in the field of using digital technologies in the management of adverse drug reactions.

In the context of the Web of Science database, the term “most productive organizations” refers to institutes, universities, or research organizations that show high productivity in the publication of scientific research. Productivity is estimated based on the number of scientific articles published by these organizations in indexed journals as well as the number of citations to their publications by other researchers. Such assessments of the activity of organizations provide the opportunity to identify leaders in scientific activities and identify those institutions that make a significant contribution to various fields of science.

Among the organizations most active in the field of drug side effect management using digital technologies, American and Chinese universities dominate. It should be noted that the journal with the highest number of published documents is the Journal of Chemical Information and Modeling. Elsevier, Springer Nature, MDPI, Wiley, and IEEE are most active in publishing research findings on digital solutions to effectively monitor and manage unwanted drug effects.

Citation of publications is most often considered a criterion for their influence on the development of studies. The most cited publications on the use of digital technologies in the management of adverse drug reactions are presented in Table 3.

The articles presented in Table 3 were each cited more than 200 times. This indicates their high assessment by the world scientific community and the presence of a scientific discussion confirming the relevance of the topic under study. The first most-cited article has an overview value in relation to the use of molecular docking in the pharmaceutical field for virtual screening and pre-selection of potential drug compounds, taking into account the prediction of adverse drug reactions and drug repurposing [15]. The possibility of a combination of molecular docking and artificial intelligence is noted. The second article highlights the possibilities of using electronic health records for clinical research [16]. The third article details meta-analysis conducted on the efficacy and safety of berberine in the treatment of type 2 diabetes mellitus, hyperlipemia, and hypertension using digital databases. No serious side effects of berberine have been reported [17]. The fourth paper is a review publication on the prediction of epileptic seizures, noting the possibility of reducing the side effects of antiepileptic drugs using statistical analysis of EEG signals and intelligent engineering systems [18]. The fifth article examines the benefits of repositioning approved drugs with a network-based deep-learning approach (deepDR) [19].

Research on the management of adverse drug reactions using digital technologies includes a variety of approaches, reflecting the broad interest and desire for innovation in the field.

The top 20 keywords by frequency of occurrence in the field of using digital technologies in the management of adverse drug reactions are presented in Table 4.

Data from Table 4 suggest that studies in the management of adverse drug reactions using digital technologies are broad, and include various methods aimed at predicting, identifying, and ensuring the safety and efficacy of drugs, as well as discovering new drugs and identifying potential compounds.

**Figure 3:**
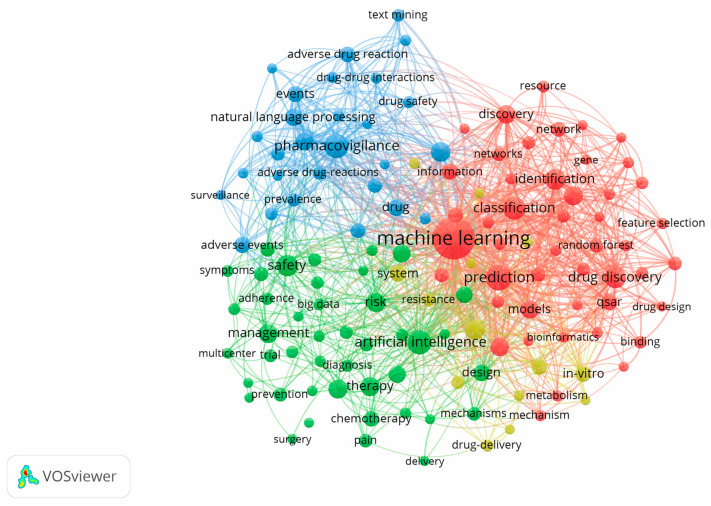
Keyword map of publications in the field of using digital technologies in the management of adverse drug reactions in Web of Science (1991–2023).

The resulting larger array of keyword data was visualized using VOSviewer 1.6.18. The keyword map shows the frequency of use of terms (the size of the circle), the strength of connections between them (the closer, the stronger), and different combinations of terms both within clusters and between them. Based on the results of the analysis, we obtained four clusters (Figure 3).

The first cluster (red) is associated with preclinical drug studies. This cluster includes the following major keywords: database, discovery, docking, drug design, drug discovery, drug repositioning, drug repurposing, hepatotoxicity, induced liver injury, machine learning, molecular docking, pharmacology, QSAR (Quantitative Structure-Activity Relationship), virtual screening, etc.

The second cluster (green) is associated with clinical trials of the drug. The following major keywords can be attributed to this cluster: adherence, artificial intelligence, big data, digital health, double-blind, drug-drug interaction, efficacy, meta-analysis, multicentral, outcomes, prevention, risk, safety, adverse effect, trial, etc.

The third cluster (blue) is associated with the pharmacovigilance of drugs. It covers the following keywords: adverse drug event, adverse drug reaction, COVID-19, data mining, deep learning, drug safety, electronic health record, natural language process, pharmacovigilance, social media, surveillance, Twitter.

The fourth cluster (yellow) is associated with a reduction in adverse drug reactions to improve the patient’s quality of life. It includes the following keywords: cancer, drug delivery, neural network, support vector machine, resistance, etc.

## 3. Discussion

There are different classifications of adverse drug reactions, according to which they are distinguished by the pathogenesis of occurrence, systemic manifestations, severity, frequency of occurrence, prognosis, etc. However, today, WHO and most countries use a classification that includes six types of adverse drug reactions [20].

Type A adverse drug reactions are dose-related adverse reactions. Most often, this type of ADR occurs when the dosage regimen is violated and the use of drugs in amounts exceeding the maximum therapeutic doses occurs. Adverse reactions of type A are characterized by low mortality. The risk of their development is high in elderly people, patients with renal or liver failure, and patients exposed to polypharmacy. They refer to preventable side reactions.

Type B adverse drug reactions are non-dose-related reactions. This type of reaction includes immunoallergic, genetically determined, and unknown reaction mechanisms. They occur less often, are often serious, and are difficult to foresee. They are characterized by high mortality.

Type C adverse drug reactions are reactions resulting from long-term therapy. Reactions of this type include withdrawal syndrome, drug dependence, cumulative effects and effects of suppressing hormone production, and tolerance. Reactions of this type are often regarded as severe, capable of significantly affecting human health, and, by their nature, irreversible by the time they are detected.

Type D adverse drug reactions are time-related reactions. They include carcinogenic, mutagenic, and teratogenic effects, reproductive defects, and many others that can occur months or years after treatment. There are also reactions associated with drug withdrawal (type E) and failure of therapy (type F).

The management of adverse drug reactions depends on their type. Prevention of type A reactions is decided on an individual patient-physician level. The main preventive measures for this type of reaction are the education of physicians in drug therapy safety and the identification of patients with risk factors. Preventive measures for type A reactions also include additional warnings in the labeling of medicines, including those that are well known to physicians and have been on the pharmaceutical market for a long time.

Management of type B reactions involves mainly regulatory measures such as labeling cautions, restrictions on use, or license suspension. Prevention of type B reactions at the individual level is virtually impossible.

Thus, the management of adverse drug reactions can be carried out both at the level of the individual patient and the general population, including through the use of digital technologies. At the individual level, it is aimed at preventing an adverse reaction in a particular patient or reducing adverse effects in the event of its development. Adverse drug reaction management at the population level includes regulatory measures. The basis for these measures is most often spontaneous reports of adverse reactions by health professionals. Spontaneous reports are particularly important for the detection of type B reactions because they occur at such a low frequency that they are difficult to detect by other methods.

We reviewed the possibilities of identifying adverse drug reactions using digital technologies within the framework of the identified four clusters: “preclinical studies”, “clinical trials”, “pharmacovigilance”, and “reduction of adverse drug reactions in order to improve the patient’s quality of life”.

### 3.1. Preclinical Drug Studies

Within the framework of the publications of the first cluster, “preclinical drug studies”, using digital technologies, including artificial intelligence and machine learning, scientists determine promising directions in the creation of new medicines [21,22,23]. It is worth noting that the use of digital technologies significantly reduces the time and cost of developing new drugs.

As previously noted, the most cited publication related to the “preclinical drug studies” cluster is Pinzi, L. et al. [15]. Researchers note the possibilities of molecular docking combined with artificial intelligence to predict side effects and repurpose drugs.

The creation of innovative drugs is associated with the need to study the interaction of many substances with molecular targets. A rational approach to the search for new, safer, and more effective drugs can be realized only on the basis of the assessment of the biological activity of compounds by computational methods.

For example, to optimize the drug search process, virtual screening techniques are used. Virtual screening allows researchers to select the most promising molecules using computer modeling [24]. The method allows assessment of the potential effect of molecules (ligands) on biological targets (mainly proteins) involved in the development of the disease. The concept of virtual screening includes the construction of molecules based on known structures of compounds with established activity (structure-based design) and the creation of molecules based on knowledge of the biotarget structure (target-based design).

Virtual screening has two aspects: reducing the cost of the process, and the possibility of targeted drug creation to influence a specific biotarget for the treatment of a particular disease. Work on molecular screening has become possible with the development of artificial intelligence technologies, which make it possible to process huge amounts of data and find non-obvious correlations in the system: descriptors and activity.

Currently, many researchers are working intensively to replenish and systematize information on the biological activity of chemical compounds, both by developing existing databases and forming new specialized information resources on drugs. Thus, Daina, A. et al. systematized open-access databases and datasets for computer drug design [25].

During the preclinical phase, studies are conducted to assess not only the efficacy but also the safety of drugs. It is important to identify potential adverse drug reactions and their mechanisms. At this stage, steps can be taken to reduce risks by, for example, modifying the structure of the drug or selecting safer candidates [26]. So, Lee, C.Y. et al. presented an overview of methods for detecting and categorizing side effects by applying deep learning approaches [27]. An in vitro machine learning-based system is reported to identify seizure-inducing side effects prior to clinical trials [28]. Pérez Santín, E. et al. review various AI methodologies used to predict drug toxicity [29].

A number of studies focus on predicting hepatotoxicity, cardiotoxicity, and drug carcinogenicity using machine learning and deep learning models [30,31]. Li, X. et al. investigate the effectiveness and accuracy of chemical-based approaches in predicting long-term toxic effects [32]. The use of artificial intelligence to predict interactions between drugs is also noted, which can significantly improve the safety and effectiveness of medical treatment [33]. The use of digital twins at the stage of preclinical research is also of interest [34,35].

Another way that artificial intelligence contributes to the creation of safe drugs is by identifying new indications for existing drugs. Drug repurposing means the use of a known medicinal product, successfully used in the treatment of one disease, for the prevention and treatment of another pathological process [36]. In other words, these are new indications for the use of a proven drug or its combination with other known substances to enhance the activity of the drug or reduce adverse drug reactions. For example, initially, dapagliflozin was developed to treat type 2 diabetes, but today it is also used to treat heart failure and chronic kidney disease. With its ability to identify patterns in large data sets that are not so obvious to researchers, artificial intelligence is useful for making possible connections between drugs and pathologies and identifying new uses for drugs that are already on the market.

The use of digital technologies at the level of preclinical drug studies significantly improves the preliminary assessment of drugs aimed at reducing side effects. Molecular design technologies are used to create more accurate and effective molecular structures for drugs. This makes it possible to reduce the likelihood of interaction with undesirable biological targets, which reduces the risk of side effects. Virtual toxicity modeling provides an opportunity to simulate drug exposure to biological systems without the use of animals. This speeds up the toxicity assessment process and provides more accurate data on potential side effects. Data integration allows for the collection and analysis of diverse data from a variety of sources, such as biochemical and genetic data, clinical trial results, and molecular structure information. This provides a comprehensive view of the potential risks and benefits of medicines. The use of digital technologies to repurpose drugs may include reducing dosages to minimize side effects. These technologies allow researchers and pharmacists to pre-identify potential drug problems and make adjustments at the development stage, thereby reducing the likelihood of undesirable adverse reactions in subsequent use by patients.

The complexity of drug development is increasing due to the constant increase in requirements for their safety and effectiveness. Literature data indicate that a rational approach to the search for new, safer, and more effective drugs can be implemented based on assessing the biological activity of compounds using digital technologies at the stage of preclinical research.

Thus, scientists use machine learning algorithms and artificial intelligence-based tools to simplify drug discovery and development processes, in a timely manner at the stage of the preclinical study of drugs, to prevent undesirable adverse reactions and thereby improve their safety. Experts note that in the future, the use of artificial intelligence can significantly reduce the development time of the drug, increase the chances of success based on the results of clinical trials, and more effectively repurpose drugs by identifying new molecules.

### 3.2. Clinical Trials

As part of the clinical studies stage, the safety of the drug is actively monitored in various groups of patients. Changes in dosage, treatment protocols, or even discontinuation of development are possible if serious adverse drug reactions are found. However, it should be noted that due to the limited sample size in scientific studies, adverse drug reactions may not be detected until after the drug is introduced to the market.

Artificial intelligence algorithms can analyze huge databases of molecular structures, biological properties, and clinical trial results to identify potential targets for new drugs and predict their efficacy and adverse drug reactions.

AI helps the clinical trial process in three key ways: it makes the process faster, more reliable, and safer. AI-enabled software is much better at finding coding errors and tuning calculations. This allows researchers to focus on more important aspects of the study, those aspects that particularly need human intervention to manage the drug during the clinical trial process.

Highly cited publications related to the cluster “clinical studies” include the paper of Madla, C.M. et al. [37]. The authors emphasize the relevance of implementing machine learning algorithms to optimizing drug development, taking into account differences in adverse reactions, efficacy, and pharmacokinetics in men and women.

Cascini, F. et al. reviewed the landscape of artificial intelligence applications in clinical studies [38]. The authors conclude that there is potential for the introduction of artificial intelligence in clinical trials. The need for further research was noted.

Pharmaceutical companies are actively introducing modern digital technologies in the development of medicines. For example, AstraZeneca is launching Health-Tech’s “Evinova” division to speed up clinical trials with AI [39].

The development of a machine learning system to predict adverse drug reactions at the clinical research stage has been reported. Galeano, D. et al. tested a system for 505 therapeutically diverse drugs and 904 side effects from different human physiological systems [40]. It has been demonstrated that a data integration strategy can predict adverse drug reactions.

The effectiveness of the use of machine learning methods in the phase I clinical study of irinotecan in patients with metastatic colorectal cancer is noted [41]. The researchers identified markers (hemoglobin, serum glutamine oxaloacetic transaminase, and albumin) significantly associated with irinotecan toxicity.

The use of digital technologies in compassionate application programs is of interest. Such programs allow the use of an unregistered drug product for sympathetic reasons for a group of patients with a chronic and serious disabling or life-threatening disease whose satisfactory treatment with a permitted drug product is impossible. Greenbaum D. et al. propose the use of digital twins to analyze the results of studies of patients participating in compassionate use programs [42].

Thus, artificial intelligence and machine learning (AI) play an important role in improving clinical trial processes and the timely detection of adverse drug reactions. For example, machine learning algorithms are able to analyze large amounts of data and identify latent patterns associated with possible drug side effects. This allows the identification of potential risks in the early stages. Machine learning also can help optimize the design and implementation of clinical trials by highlighting more representative patient groups and minimizing the risks of side effects. In general, the need for more research is emphasized.

### 3.3. Pharmacovigilance

Information on scientific development is presented for the first time in the registration dossier when registering the drug, and then updated when obtaining new knowledge during the life cycle of the drug [43]. After the introduction of the drug to the market, monitoring of its safety and effectiveness continues. New adverse drug reactions can be detected, or the frequency of known ones can be estimated. Instructions for drug use may be updated in light of new data. The identified information on adverse drug reactions is entered in the instructions for medical use of drugs, which contributes to ensuring the safety of pharmacotherapy.

The introduction of information technology is necessary to improve the pharmacovigilance system. Scientists highlight the following digital pharmacovigilance approaches: electronic databases of spontaneous reports, monitoring of electronic medical records, social networks, and analysis of digital device data [44,45].

Electronic spontaneous reporting databases are a key source for detecting adverse reaction signals, playing an important role in detecting information about possible drug safety issues [46].

VigiBase is a database of adverse drug reactions that is part of the World Health Organization’s (WHO) International Adverse Drug Reaction Monitoring Program [47]. This database contains information from various national pharmacovigilance centers worldwide. VigiBase is used to analyze and monitor the safety of drugs, as well as to identify new or rare adverse reactions to drugs. Data from VigiBase assist scientists, medical professionals, and regulatory authorities in assessing drug safety and taking appropriate pharmacovigilance measures.

The results of studies of agranulocytosis and anti-neutrophil cytoplasmic antibody-associated vasculitis caused by methimazole and propylthiouracil drawing on data from Vigibase are of interest. The findings can serve as a basis for improving treatment strategies, improving the safety of drug therapy, and optimizing care for patients with thyroid disease [48].

In parallel with the use of a common adverse reaction database, each country created and improved its own system for collecting and analyzing adverse drug reactions (for example, Critical incident report system (Austria), Database of Adverse Event Notification (Australia), French PharmacoVigilance Database, FDA Adverse Event Reporting System (USA), MedEffect (Canada), PHARMO (The Netherlands), etc.).

EudraVigilance currently plays a key role in monitoring drug safety in the European Union. This system records and analyzes adverse effect reports from medical professionals, patients, and drug manufacturers. This information allows clinicians to assess and manage the risks associated with the use of medicines and make appropriate decisions on their safety.

The functioning of international databases, therefore, significantly increases the ability to exchange experience and information in the field of drug safety, and is an important part and a peculiar driver of global pharmacovigilance.

The use of digital tools for processing the results of databases of adverse reactions is promising. For example, Kiryu, Y. et al. developed a drug adverse reaction analysis system that uses machine learning to analyze the Japanese Adverse Drug Event Report (JADER) database [49]. Salas, M. et al. also note the positive impact of machine learning on pharmacovigilance processes [50].

Additional resources for screening information on ADR can be websites of regulatory agencies, clinical trial registries, scientometric databases, and social networks.

AI technologies are applicable to several areas of pharmacovigilance. In addition to efficient handling of adverse reaction reports, in pharmacovigilance, the use of digital technologies allows for the analysis of scientometric databases, tracking of information on social networks (for example, Twitter), and facilitating clinical decision-making [51,52].

The most highly cited publication of the cluster “pharmacovigilance” is the paper by Freifeld, C. C. et al. [53]. The authors note the need for more work to improve the collection of data on adverse drug reactions on Twitter.

Analysis of electronic medical records allows clinicians to take into account the main stages of treatment for patients, prescriptions, studies, assigned tests, and their results. The advantage is also the single input and reuse of the primary information received from the patients. Increasing the effectiveness of monitoring the condition of patients through the unification of methods for collecting, systematizing, storing, and analyzing information contributes to improving patient safety and reducing adverse drug reactions.

New modern electronic methods, for example, online applications or mobile applications, complement, simplify, and expand the ability to exchange and obtain important available information on drug safety, as well as improve adherence to treatment and reduce the side effects of polypharmacy [54,55,56].

The development of big data processing technologies, such as AI, allows clinicians to extract valuable clinical information from accumulated electronic medical records and use it both to collect data on adverse drug reactions and to make medical decisions [57].

Reducing human involvement in day-to-day data collection, capture, validation, and analysis operations reduces the likelihood of errors and improves the quality and accuracy of results.

The use of data from social media and digital devices allows active monitoring of patient feedback and reports of side effects. This feedback enriches the database and enables continuous monitoring.

Machine learning algorithms in pharmacovigilance systems can automatically analyze and filter adverse event data, enabling more effective monitoring and response to abnormalities.

Thus, the extraction of information using AI, machine learning, and other digital technologies makes it possible to assess emerging adverse drug reactions during pharmacovigilance.

### 3.4. Reduction of Adverse Drug Reactions in Order to Improve the Patient’s Quality of Life

Publications in the fourth cluster are related to the use of digital technologies to reduce adverse drug reactions in order to improve the patient’s quality of life.

The adverse drug reactions may be unavoidable, i.e., foreseen in advance, but the beneficial effects conventionally outweigh the potential harms. For example, the efficacy of rifampicin against tuberculosis exceeds its potential hepatotoxicity. The antimicrobial effects of aminoglycosides far outweigh their potential adverse effects on the kidneys and hearing. In this context, digital technologies can contribute to the monitoring and management of adverse drug reactions. For example, the use of e-health systems allows for more efficient tracking of patient data, more accurate dosage selection, and rapid detection of adverse drug reactions. Digital technologies can also be used to create drug toxicity prediction models, which can predict likely risks based on individual patient characteristics. Such innovations help to optimize treatment, minimize adverse drug reactions, and improve the safety of drug use.

Using AI, healthcare providers can predict the likelihood of developing certain side effects in particular patients, which allows measures to be taken in advance to prevent or manage them. Kim, Y. et al., using machine learning methods, predicted neutropenia in 48 h in breast and lung cancer patients prescribed cytotoxic drugs. The results obtained make it possible to identify patients with an increased risk of neutropenia and make an appropriate medical decision [58]. Other authors have proposed a model for predicting the risk of bleeding with rivaroxaban, which will facilitate the individualized treatment of geriatric patients [59].

The results of these publications suggest that artificial intelligence can analyze a patient’s genetic information and provide more personalized treatment regimens while minimizing negative side effects [60]. Chi, C.L. et al. propose using machine learning in big data processing to develop personalized medical treatment plans to prevent side effects in patients taking statins [61].

AI systems can process data from a variety of sources, including medical records, wearables, and sensors, to monitor physiological parameters and detect changes related to side effects, physician decision-making, and dose correlation.

A digital sensors review for non-invasive drug monitoring belongs to the highly cited publications of Cluster Four. The authors note that digital sensors contribute to the development of personalized medicine, increase the effectiveness of therapy, and reduce side effects [62].

IoMT-based drug tracking allows physicians to monitor the effect of prescribed drug dosages on a patient’s condition. Digital communication and reminder systems for physicians can significantly improve the safety and effectiveness of drug use. In turn, patients can monitor medication using reminders and mark on the app how their symptoms change for further analysis by a doctor. In addition, digital applications can provide patients with educational materials about the impact of diet changes and habits (e.g., skipping or delaying breakfast) on the effectiveness and safety of drugs.

Analysis of data from electronic medical records and spontaneous report databases allows the creation of models predicting how different groups of patients may respond to drug exposure. This helps personalize the treatment approach and prevent side effects in exposed groups.

Detection of type A–D adverse reactions, including the use of digital technologies, begins in the early stages of preclinical studies and continues in the subsequent stages of drug development and use.

Thus, the use of digital technologies, including artificial intelligence, in the medical field has the potential to significantly improve the prediction, prevention, and management of adverse drug reactions, contributing to a more effective, safe, and personalized approach to medical treatment.

It is worth noting that the introduction of digital technologies in medicine entails the collection, storage, and processing of large amounts of patient personal data. This poses potential risks to data privacy and security, requiring strict measures to protect against unauthorized access, information leaks, and abuse of personal data.

In addition, the introduction of new digital technologies requires the appropriate training of medical personnel. Insufficient preparation for the use of new tools can lead to errors in data processing, misinterpretation of results, and a lack of efficacy in managing adverse drug reactions.

The results of the analysis of digital data on adverse drug reactions may be distorted or incomplete in the case of poor quality and incomplete information. This may be due to errors in data entry, a lack of uniform standards for recording information, or incomplete medical records. Insufficient reliability of the data may influence the accuracy of the analysis, leading to inaccurate conclusions and decisions in the field of pharmacotherapy. Consideration and management of these risk factors are critical aspects of the implementation of digital technologies in the management of adverse drug reactions, and require additional attention and regulation within the framework of research and practical application.

## 4. Materials and Methods

### 4.1. Database Selection

A bibliometric analysis of publications in the Web of Science database for 1991–2023 was carried out. The Web of Science database provides a wide range of analytical capabilities for document flow research. The selection of works for analysis was carried out based on the results of a search query using keywords, abstracts, and publication titles.

Each of the bibliographic databases, such as Web of Science (WoS), PubMed, and Embase, has its own unique characteristics and advantages [63]. Our study is interdisciplinary in nature because it is at the intersection of digital healthcare, pharmacy, pharmacology, and clinical medicine. In connection with the above, we chose the Web of Science database. The Web of Science is a big multi-sectoral database covering various areas of knowledge. This allows us to consider the problem of side effects of drugs from many points of view, including medical, pharmaceutical, technological, and other aspects. The integration of data from different disciplines provided by Web of Science can be particularly useful in studying the problem of side effects, which crosses the boundaries of medicine, pharmaceuticals, and technology. The use of the Web of Science database may provide a broader scope of research. Moreover, the Web of Science provides extensive tools for bibliometric analysis, including citation statistics, influence factors, and other metrics. Naturally, the Web of Science may have many scientific articles that are also available in other database such as PubMed.

### 4.2. Keywords Selection

The keywords in this study were chosen in order to cover the topic of the use of digital technologies in managing adverse drug reactions as accurately and comprehensively as possible. These keywords were tailored to provide broad but at the same time specific coverage of the literature on the topic, given the relevance of the study and the purpose of bibliometric analysis. The inclusion of the word “digital” is aimed at highlighting the work related specifically to the use of digital technologies. Our search strategy included the use of double quotes in the query. We were interested in analyzing articles involving, for example, the phrase “side effect” (rather than articles mentioning the words “side” and “effect” in different parts of the abstract). This applies to all phrases where we used quotation marks. Using this approach reduces the level of information noise. We used the OR operator for synonyms: “side effect*”, “adverse reaction*”, “adverse event*”, and the widely used digital tools: “machine learning*” and “artificial intelligence*”.

In the Web of Science search engine, the “*” sign means truncation of the word on the side where it is marked with this sign. Using the “NOT” operator made it possible to exclude from the array irrelevant articles where the data fields of the Web of Science system (article title, abstract, key words) contained such irrelevant terms as digitalin* or digitalis* or supplemental digital* or digital ulcer*.

The final form of the search-request was as follows:

(digital* AND (drug “adverse effect*” OR drug “adverse reaction*” OR drug “adverse event*” OR drug “side effect*”) NOT (digitalin* OR digitalis* OR “supplemental digital*” OR “digital ulcer*”)) OR (“machine learning*” AND (drug “adverse effect*” OR drug “adverse reaction*” OR drug “adverse event*” OR drug “side effect*”) NOT (digitalin* OR digitalis* OR “supplemental digital*” OR “digital ulcer*”)) OR (“artificial intelligence*” AND (drug “adverse effect*” OR drug “adverse reaction*” OR drug “adverse event*” OR drug “side effect*”) NOT (digitalin* OR digitalis* OR “supplemental digital*” OR “digital ulcer*”)).

### 4.3. Data Analysis

As of 31 December 2023, 1241 publications from the Web of Science database have been selected for analysis. Methods of informational and bibliographic search, system approach, methods of analysis, and generalization were used. For the analysis, the authors used a bibliometric tool, specifically VOSviewer 1.6.18.

The Web of Science database provides scientists with extensive opportunities for bibliometric analysis and research of the scientific literature: search, filtering, and evaluation of citations, as well as analysis of co-authorship and network connections. No additional filtering of the obtained hits was applied in our study. We employed the Analyze Results and Citation Report features on the Web of Science platform to conduct basic frequency counts and determine the number of citations per publication (average citations per item within a specific subset) for the most prolific institutions, journals, and publishers. Subsequently, the publications (n = 1241) were exported as tab-delimited files to VOSviewer for the creation of a term map.

### 4.4. Inclusion/Exclusion Criteria (Language, Year of Publication)

The Web of Science database includes articles in various languages. However, most of the articles in this database are presented in English, as English is the dominant language in scientific publications around the world. The Web of Science database allows researchers to search in several languages. We used English keywords. Our study includes publications that have only English titles and annotations, although they are published in other languages: German (n = 7), Japanese (n = 5), French (n = 4), Chinese (n = 2), Portuguese (n = 2), Russian (n = 2), and Czech (n = 1).

The results of our analysis revealed publications in the Web of Science since 1991. In this context, it should be noted that significant technological changes occurred in the 1990s, including improvements in the fields of databases and computer technology. Thus, the choice of 1991 as a start date presupposes a balance between historical perspective and modern relevance, providing a focus on the last thirty years of development in this field.

## 5. Conclusions

An analysis of the dynamics of publications in the field of the use of digital technologies in the management of adverse drug reactions for the period from 1991 to 2023 indicates its positive, exponential nature. A wide geographic distribution of institutions conducting research on the topic under study has been identified. 72% of all publications originate from the following countries: the USA, China, England, India, and Germany. The majority of reports (32%) are from the USA. The most productive organizations, journals, and publishers publishing articles in this area have been identified.

According to the results of keyword analysis using VOSviewer 1.6.18, four clusters were obtained in the field of using digital technologies in the management of adverse drug reactions, which are closely related to the stages of preclinical and clinical trials, the pharmacovigilance system, and the reduction of adverse drug reactions to improve the patient’s quality of life.

Digital technology applications at the preclinical research stage include the following areas: molecular design technologies, virtual toxicity simulation models, data integration, drug repurposing, etc., aimed at improving both the efficacy and safety of medicines.

Integration of the use of machine learning algorithms to analyze data monitoring in electronic databases of spontaneous reports, electronic medical records, scientometric databases, social networks, and data analysis of digital devices both at the stage of clinical trials and in the pharmacovigilance system is also aimed at achieving efficacy, safety of medicines, early detection of undesirable adverse reactions, possible prevention of them, and improving the quality of life of patients.

The results of combining the above-mentioned digital technologies from all four clusters provide a huge synergy of relevant and valuable information for health professionals, patients, health authorities, regulators, and pharmaceutical companies. The study of bibliometric data allows healthcare professionals to identify current topics and trends in digital technology to manage drug side effects. Patients using digital technology in treatment can be more involved in their health decisions and interactions with medical professionals. Health authorities and pharmaceutical companies can use the respective data to develop strategies and policies for managing adverse drug reactions using digital technologies, allocating funding for innovative research, and implementing new technologies.

It is worth noting some risk factors for the use of digital technologies in the management of adverse drug reactions: personal data management, staff training, quality, and completeness of information.

The dissemination of reliable medical information, the study of the benefits and risks of drugs, and the introduction of digital technologies into pre-clinical and clinical practice will significantly improve the quality of patient care and the safety of pharmacotherapy.

### Limitations

This study had several limitations. Limitations of the study include focus on the Web of Science database only, use of certain keywords and operators in search queries, limitation to English keywords only, and time interval since 1991. For more comprehensive analysis, future research may include integrating data from other databases (such as PubMed and Embase), expanding the list of keywords, reviewing work in different languages, and a wider time range. These steps will contribute to a deeper understanding of the topic.

## Figures and Tables

**Table 1:** Distribution of publications by document type in the field of using digital technologies in the management of adverse drug reactions in Web of Science (1991–2023).

Document Types	N
Article	889
Review article	250
Proceedings paper	102
Editorial material	9
Meeting Abstract	5
News Item	1

**Table 2:** Top five most productive affiliations, journals, and publishers.

Entity	Record Count	(% of 1241)
Affiliations		
University of California System	34	2.74
Harvard University	26	2.10
Stanford University	24	1.93
Chinese Academy of Sciences	21	1.69
Pennsylvania Commonwealth System of Higher Education	20	1.61
Journals		
Journal of Chemical Information and Modeling	22	1.77
Journal of Medical Internet Research	20	1.61
Frontiers in Pharmacology	19	1.53
Briefings in Bioinformatics	17	1.40
Drug Safety	17	1.40
Publishers		
Elsevier	213	17.16
Springer Nature	206	16.60
MDPI	91	7.33
Wiley	89	7.17
IEEE	66	5.32

**Table 3:** The top five most cited publications by authors studying digital technologies in the management of adverse drug reactions in the Web of Science (1991–2023).

Title	Year of Publication	Average per Year	Total Citations
Molecular Docking: Shifting Paradigms in Drug Discovery	2019	608.3	20,074
Clinical information extraction applications: A literature review	2018	113	678
Meta-analysis of the effect and safety of berberine in the treatment of type 2 diabetes mellitus, hyperlipemia and hypertension	2015	48.43	339
Prediction of epileptic seizures	2002	29.8	298
deepDR: a network-based deep learning approach to in silico drug repositioning	2019	12.26	282

**Table 4:** The top 20 keywords in the field of using digital technologies in the management of adverse drug reactions in Web of Science (1991–2023).

Keywords	Occurrences
Machine learning	299
Prediction	92
Pharmacovigilance	81
Artificial intelligence	75
Safety	58
Drug discovery	58
Identification	56
Deep learning	50
Cancer	49
Therapy	45
Management	45
Risk	45
Efficacy	44
Database	44
Model	43
Discovery	42
Drugs	39
Social media	38
Natural language processing	37
Double-blind	35

## Data Availability

Data is contained within the article.
